# Altered movement patterns and muscular activity during single and double leg squats in individuals with anterior cruciate ligament injury

**DOI:** 10.1186/s12891-015-0472-y

**Published:** 2015-02-13

**Authors:** Anna Trulsson, Michael Miller, Gert-Åke Hansson, Christina Gummesson, Martin Garwicz

**Affiliations:** Department of Health Sciences, Physiotherapy, Lund University, Lund, Sweden; Department of Rehabilitation Medicine, Skane University Hospital, Lund, Sweden; Occupational and Environmental Medicine, Lund University, and University and Regional Laboratories Region Scania, Lund, Sweden; Centre for Teaching and Learning, Lund University, Lund, Sweden; Department of Experimental Medical Science, Neuronano Research Center, Lund University, Lund, Sweden

**Keywords:** Anterior cruciate ligament, Movement pattern, Muscular activity, Motor skills, Motor control, Single leg squat, Electromyography (EMG), Postural orientation, Assessment, Physiotherapy

## Abstract

**Background:**

Individuals with Anterior Cruciate Ligament (ACL) injury often show altered movement patterns, suggested to be partly due to impaired sensorimotor control. Here, we therefore aimed to assess muscular activity during movements often used in ACL-rehabilitation and to characterize associations between deviations in muscular activity and specific altered movement patterns, using and further exploring the previously developed Test for substitution Patterns (TSP).

**Methods:**

Sixteen participants (10 women) with unilateral ACL rupture performed Single and Double Leg Squats (SLS; DLS). Altered movement patterns were scored according to TSP, and Surface Electromyography (SEMG) was recorded bilaterally in six hip, thigh and shank muscles. To quantify deviations in muscular activity, SEMG ratios were calculated between homonymous muscles on injured and non-injured sides, and between antagonistic muscles on the same side. Correlations between deviations of injured/non-injured side SEMG ratios and specific altered movement patterns were calculated.

**Results:**

Injured/non-injured ratios were low at transition from knee flexion to extension in quadriceps in SLS, and in quadriceps and hamstrings in DLS. On injured side, the quadriceps/hamstrings ratio prior to the beginning of DLS and end of DLS and SLS, and tibialis/gastrocnemius ratio at end of DLS were lower than on non-injured side. Correlations were found between specific altered movement patterns and deviating muscular activity at transition from knee flexion to extension in SLS, indicating that the more deviating the muscular activity on injured side, the more pronounced the altered movement pattern. “Knee medial to supporting foot” correlated to lower injured/non-injured ratios in gluteus medius (r_s_ = −0.73, p = 0.001), “lateral displacement of hip-pelvis-region” to lower injured/non-injured ratios in quadriceps (r_s_ = −0.54, p = 0.03) and “displacement of trunk” to higher injured/non-injured ratios in gluteus medius (r_s_ = 0.62, p = 0.01).

**Conclusions:**

Deviations in muscular activity between injured and non-injured sides and between antagonistic muscular activity within injured as compared to non-injured sides indicated specific alterations in sensorimotor control of the lower limb in individuals with ACL rupture. Also, correlations between deviating muscular activity and specific altered movement patterns were suggested as indications of altered sensorimotor control. We therefore advocate that quantitative assessments of altered movement patterns should be considered in ACL-rehabilitation.

**Electronic supplementary material:**

The online version of this article (doi:10.1186/s12891-015-0472-y) contains supplementary material, which is available to authorized users.

## Background

Altered movement patterns in individuals with Anterior Cruciate Ligament (ACL) injury have been demonstrated during gait, functional movements and common rehabilitative exercises [1-3]. In clinical practice of ACL injury evaluation and rehabilitation, assessments primarily focus on knee stability, muscular strength and/or knee symptoms [4-6], and since it has been argued that interventions should be based on functional performance tests [7], assessments of jump performance are also often used [8]. Still, none of these tests strongly predict the demonstrated alterations in movement patterns [9,10].

The underlying mechanisms to altered movement patterns in individuals with ACL injury, are to date not fully understood, but impaired sensorimotor control – including delayed muscular responses, altered timing, co-activation or proprioception – likely plays an important causal role [2,11]. Indeed, altered afferent sensory input due to joint injury and altered information processing in the central nervous system can lead to altered efferent output to muscles [12,13]. In addition, individuals with ACL injury demonstrate biomechanical instability due to increased knee-joint laxity between tibia and femur, with increased anterior-posterior translations and/or internal-external rotation, which may increase the risk of “giving-way” episodes (abnormal, sudden, painful displacement of tibia relative to femur during weight bearing) [14].

To provide a better understanding of the interplay between alterations in sensorimotor control and biomechanical joint stability following knee injury, and to further explore the previously developed Test for Substitution Patterns (TSP) for the assessment of alterations in functional movements and common rehabilitative exercises, we investigated movement patterns and muscle activity in individuals with ACL rupture. Since anticipatory postural muscular activity can be altered in individuals with ACL injury [13,15], the notion was that specific altered movement patterns are associated with deviations in muscular activity between injured and non-injured sides. If there is such association, it could have implications for future rehabilitation guidance where detection and improvement of altered movement patterns associated with deviations in muscular activation should be considered in ACL rehabilitation.

To assess altered movement patterns, reliable, valid and preferably quantitative observational assessments of movement patterns are needed. In this study we focused on the movements Single Leg Squat (SLS) and Double Leg Squat (DLS), since these movements have previously been used in standardized assessments in individuals with knee-complaints, have proved to be both valid and reliable and are often used as functional rehabilitative exercises [16-19]. During SLS and DLS, altered movement patterns have been observed as poor alignment manifested as excessive medial displacement of the knee in relation to the foot or hip, an alignment that is reported to be especially disadvantageous in individuals with ACL injury since it can cause an increased risk for re-injuries during “giving-way” episodes [20]. Moreover, the SLS and DLS can be used in knee rehabilitation not only for evaluation, but also as a functional weight-bearing training task to improve neuromuscular control [21].

However, assessments of SLS and DLS have in different studies been performed in somewhat different ways; either dichotomously or on an ordinal scale for segmental or overall movement quality [16,18,19,22]. Furthermore, poor alignment in the lower extremity is not dependent only on knee joint position, but also on postural orientation of other joints in the lower leg and/or trunk (the ability to stabilize body segments in relation to each other and to the environment [23]), maintained by the dynamic multi-joint stabilization [13]. It has therefore been suggested that the total kinetic chain, from foot to neck, should be considered during these movements, and various tests have been developed for this purpose [3,9,18]. In this study, we used the Test for Substitution Patterns, since the TSP contains the SLS and DLS and has been evaluated for assessment of altered movement patterns in the entire kinetic chain in individuals with ACL injury and in controls [3,9]. In previous studies of TSP, individuals with ACL injury showed more and/or more pronounced altered movement patterns around the injured knee-joint, adjacent joints and in the trunk as compared to non-injured controls, and the TSP assessment was found to have a good inter- and intra-rater reliability at group level [3,9]. The SLS and DLS, included in the TSP, are in this context scored for specific, predefined altered movement patterns associated with knee complaints and often used also as rehabilitative exercises [24].

To understand the muscle control strategies used by individuals with ACL injury performing SLS and DLS – tasks more strenuous than gait, but still not as demanding as jump tests – we combined the assessments of altered movement patterns with assessments of muscle activity by the use of simultaneous surface electromyography (SEMG). Electromyography is often used in kinesiological studies and reflects motor unit action potentials. The technique has been used for characterization of for example dynamic joint stability and compensatory mechanisms following ACL injury [25,26]. SEMG also has been studied during SLS and DLS – although preferentially in non-injured individuals [27,28].

The aims of the present study were i) to quantitatively characterize deviations in muscular activity in six muscles of the hip, thigh and shank during SLS and DLS performance in individuals with a mechanical instability due to a unilateral, total ACL rupture, and ii) to quantitatively characterize associations between specific altered movement patterns and specific deviations in muscular activity.

## Methods

### Participants

Sixteen participants (10 women), aged 19–48 years (mean 29.5, SD 9.5) with a total, unilateral, non-reconstructed ACL rupture sustained 2–11 months (mean 3.6, SD 2.3) before testing, volunteered to participate in this cross-sectional, experimental study (Table 1). An orthopedic surgeon verified the diagnosis through clinical examination; all participants had a biomechanical knee-instability with an increased sagittal laxity indicated by a positive Lachman test and a positive pivot shift test. The injury also was verified with magnetic resonance imaging (MRI) and/or arthroscopy at the Department of Orthopedics at Lund University Hospital, Sweden, where all participants who volunteered had consulted an orthopedic surgeon during the testing period from March 2012 to January 2013. Individuals with knee-pain, injury to the contralateral knee, concomitant symptomatic or repairable meniscal injury, major cartilage injury, fracture, patello-femural injury or instability, injury to/pain in back/other joint, injury to the nervous system or a known neurological disease or pregnancy were excluded. All participants but one had regular supervised physical therapy. All participants gave their written, informed consent, and the study was approved by the Regional Ethical Review Board in Lund, Sweden, Dnr 2010/387.

**Table 1:** **Characteristics of the 16 participants with ACL rupture**

Body Mass Index (BMI; kg/m^2^), mean (SD)	24.6 (5.1)
Dominant side: right	14
Injured side: dominant	6
Participants with Magnetic Resonance Imaging-verified associated meniscal afflictions	5
collateral ligament injury	5
compressive trauma/bone bruise	13
KOOS sport/recreation, mean (SD)	38.1 (32.3)
KOOS quality of life, mean (SD)	44.7 (21.1)
IKDC, mean (SD)	58.2 (16.3)
Tegner activity scale before injury, median (min – max)	6 (3 – 10)
Tegner activity scale at the testing occasion, median (min – max)	3 (1 – 6)
TSP Total score non-injured side, median (min – max)	0 (0 – 5)
TSP Total score injured side, median (min – max)	9.5 (2 – 20)
p-value	p ≤ 0.000
median difference, 95% CI	6.5 (5 – 12)

KOOS = Knee Injury and Osteoarthritis Outcome Score. The 2 subscales sport/recreation and quality of life (range from 0 “extreme problems” to 100 “no problems”) were used in this study.

IKDC = the International Knee Documentation Committee Subjective Knee Evaluation Form (ranging from 0 “absence of symptoms” to 100 “no limitations of daily living”).

Tegner activity scale (ranging from 1 “low activity like walking on even ground” to 10 “high activity like American football”).

TSP = Test for Substitution Patterns (comprising 5 test-movements; total score ranging from 0 “no substitution pattern” to 54 “very clearly present, subject performed very poorly” where altered movements (substitution patterns) were scored as observable, predefined deviations of postural orientation).

SD = Standard Deviation.

95% CI = 95% Confidence Interval.

### Settings and procedure

The participants were informed which test-movements to perform, but not what was observed in the TSP. All were allowed to try the test-movements before the electrodes and electrogoniometers were applied. They were asked to perform the test-movements at a standardized pace given by a Metronome (50 beats per minute; Joyo JM-65, Joyo Technology CO., LTD, Shenzhen, China). The participants performed the test-movements barefooted and dressed in shorts and T-shirt. During the SLS, unilateral fingertip support was permitted. The examiner gave standardized verbal and visual instructions according to the manual, and scored participants’ performances during three trials at the same time as the SEMG and electrogoniometer measurements were recorded. After performing the test-movements, participants rated their knee symptoms, knee-function and activity level. The test session lasted about 90 minutes.

#### Test-movements and TSP-scoring

The test-movements primarily used in the present analysis were SLS and DLS, since these movements can be assessed in a reliable manner [16-19], are commonly used in both clinical work and in research, resemble activities of everyday life and are also used as common functional rehabilitative exercises. However, the complete TSP consists of five test-movements – SLS, DLS, forward lunge, tip-toe standing knee flexion and body-weight-altering, which were all conducted to assess TSP Total score (Table 1). The participants performed all five test-movements in a standardized order, with three consecutive trials of each test-movement [3,9].

The altered movements (substitution patterns) scored in the SLS and DLS were scored according to a strict protocol presented and evaluated by Trulsson et. al. [3,9], and were observable, predefined deviations of postural orientation between the foot, knee, hip, trunk, arms and/or neck as compared to the non-injured side; “knee medial to supporting foot”, “pronation of foot”, “lateral displacement of hip-pelvis region”, “displacement of trunk” in the SLS and “displacement of body weight to either side” in the DLS. Substitution patterns were scored for each side separately using a four point, ordinal scale (0–3); “0”: no substitution pattern; “1”: substitution pattern possibly present; “2”: substitution pattern clearly present; “3”: subject performed very poorly (e.g. with no similarity to the task or unable to perform the predefined number of trials). Points 1–3 were given when a specific substitution pattern was observed in at least two of the three trials of the test-movement. Consequently, in the present study, the possible score for an individual participant was 0 to 12 points for SLS and 0 to 3 points for DLS. An experienced physical therapist specializing in knee injury rehabilitation and familiar with TSP assessments scored all participants’ performances.

#### Outcome measures

The participants rated their knee symptoms as perceived during the past week and also retrospectively, as experienced just before the injury. The Swedish version of Knee Injury and Osteoarthritis Outcome Score, KOOS [4], a self-administered, validated questionnaire with 42 questions comprising five subscales, each ranging from 0 (extreme problems) to 100 (no problems) was used. The subscales sport/recreation and quality of life were used in this study (Table 1). The participants also rated their knee-function on the International Knee Documentation Committee Subjective Knee Evaluation Form (IKDC), consisting of 10 questions about symptoms and activity ranging from 0 to 100, where 100 indicates absence of symptoms and no limitations in daily living [29]. Participants rated their activity level, according to Tegner activity scale graded from level 1 (low activity) to 10 (very high activity) [30]. Before injury, median activity level was 6 (activities such as badminton, tennis, alpine skiing, aerobics or cross country running). At the test occasion median activity level was 3 (bike riding, swimming or golf).

#### SEMG

To evaluate which muscles, electrode placements, procedure and pace to use when performing the test-movements, piloting with SEMG of 13 different muscles and protocol development was carried out in two individuals with ACL rupture (not included in the study) and four non-injured individuals. In the final protocol, SEMG was recorded bilaterally from gluteus medius, biceps femoris (long head), quadriceps femoris vastus lateralis, tibialis anterior, medial head of gastrocnemius and the peroneus longus muscle, using a 16 channel telemetric data logger (Mega Muscle tester ME6000, Mega Electronics Ltd, Kuopio, Finland; sampling rate 1024 Hz). The Mega Win Software 3.1 was used to digitally filter the raw SEMG signals with a band-pass filter with cut off frequencies of 30 and 400 Hz and to calculate the root mean square value for epochs of 125 ms. Self-adhesive, silver/silver chloride surface electrodes (Ambu® Neuroline 720, Ambu, Ballerup, Denmark) were placed in a bipolar configuration longitudinally on each muscle belly (inter-electrode distance 20 mm) according to SENIAM recommendations [31]. The skin surface was prepared by shaving, if necessary, and with fine sandpaper and ethanol, and the same examiner mounted the electrodes for all participants. Before each test, SEMG signals were visually controlled during rest for background artifacts and poor electrode connection.

#### Electrogoniometers

For measurement of knee flexion/extension angle, strain gauge electrogoniometers (SG150, Biometrics Ltd., Newport, United Kingdom) with a measurement accuracy of ±2° were mounted on the lateral side of the right and left knees and signals were recorded on the same data logger as the SEMG signal. The electrogoniometers were attached with double sided medical tape, aligned so that the center of the electrogoniometer-sensor corresponded to the center of movement in the knee-joint and in a straight line from the participants’ trochanter major of femur and lateral malleolus of fibula when standing with straight legs. The electrogoniometers were calibrated according to the manufacturer’s instructions and were mounted by the same examiner for all participants.

#### Video camera

A camera (High-Definition Video 1080i; Canon HD, LEGRIA HV40, Canon Inc., Tokyo Japan), mounted on a tripod and capturing test-movements from the front, was synchronized (±40 ms) with the SEMG measurements so that the TSP examiner also could assess test-movements after testing.

### Data analysis and statistics

#### Data analysis

One examiner visually inspected the video according to a protocol defined in advance so that the trial with the most obvious altered movements was chosen for analysis. To analyze SEMG activity during the chosen trial, the Mega Win software was used to visualize SEMG amplitudes (microvolts, μV) on a time-axis (milliseconds, ms). The same examiner processed the SEMG recordings and identified the start and end of the trial by simultaneous inspection of the video and electrogoniometer recordings: the instance that the goniometer signal changed +2° during 0.1 s was defined as the start and the instance when the goniometer signal resumed starting-level was defined as the end of the test-movement. The mean durations of the SLS were 2.5 s (SD 0.5) and 2.4 s (SD 0.5) for the injured and non-injured sides, respectively and 2.6 s (SD 0.4) for the DLS.

For analysis, the SEMG-recording during test-movements was divided into 10 time epochs (bins). The time bins were evenly distributed over the duration of the test-movement, within the limits of the time resolution of the Mega Win software (0.1 sec). A “pre-bin”, bin 1, was added for the inclusion of anticipatory muscular activation just prior to the defined beginning of the test-movement, corresponding to 10% of the duration of the test-movement. Hence, 11 time bins, evenly distributed and starting slightly before the test-movement were used in the SEMG analysis.

The average amplitude for each of the 11 time bins was calculated by the Mega Win Software. However, comparisons and correlation calculations focused on preparatory muscular activity (time bin 1), the transition from flexion to extension (for individual participants either time bin 6 or 7) and the decline or cessation of muscular activity (time bin 11), given the crucial importance of these phases for the movement as a whole.

To obtain a measure of SEMG deviation – for each muscle, for each participant, for both SLS and DLS – that could be quantitatively compared to TSP scores, the ratio of average amplitude for each of the 11 time bins between injured and non-injured sides, SEMG_ratio_, was calculated in accordance with previous studies [32,33], so that equal activity of injured and non-injured sides would correspond to 1.0 [32]. To quantitatively characterize the activity in antagonistic muscles, a ratio of SEMG activity of quadriceps/hamstrings and tibialis anterior/gastrocnemius was calculated for bin 1, 6/7 and 11. Ratios were then compared between injured and non-injured sides.

### Statistics

The Wilcoxon Signed Ranks Test was used to test for differences between the injured and non-injured sides with respect to TSP scores, SEMG_ratio_ and for the antagonistic activity ratio within the injured side in bins 1, 6/7 and 11. Differences p ≤0.05 were considered statistically significant. Non-parametric confidence intervals (95%) were calculated for the median of the paired differences [34]. For the correlation calculations between the SEMG_ratio_ and the results of the TSP, the Spearman’s correlation coefficients (r_s_) were used, and correlations r_s_ < −0.5 or r_s_ ≥ 0.5 are presented. All calculations and statistical analyses were carried out using SPSS version 11.5 and IBM SPSS Statistics version 20.0.

Data for the dominant/non-dominant leg were pooled in accordance with previous studies, in which subjects conducting movements similar to SLS and DLS were found to have a fairly symmetrical SEMG when comparing dominant and non-dominant sides [35].

## Results

### TSP-score

In accordance with previous studies, TSP scores were significantly higher on participants’ injured side as compared to their non-injured side, for both SLS and DLS (Table 2). The two most frequent substitution patterns in SLS were “knee medial to the supporting foot” and “displacement of trunk” (both present in 9/16 participants). In DLS, 13/16 participants “displaced the body weight to non-injured side”.

**Table 2:** **Median TSP score for the Single Leg Squat (SLS) and Double Leg Squat (DLS)**

**Test-movement**	**Median (min – max) TSP score on injured side**	**Median (min – max) TSP score on non-injured side**
SLS	2 (0 – 6)	0 (0 – 3)
Possible score: 0 – 12	median difference 1.0, 95% CI (1.0 – 3.0)	
	p = 0.003	
DLS	1 (0 – 2)	0 (0 – 0)
Possible score: 0 – 3	median difference 1.0, 95% CI (1.0 – 2.0)	
	p = 0.001	

Median (min – max) TSP score for the Single Leg Squat (SLS) and Double Leg Squat (DLS) respectively, as well as median difference with 95% confidence interval (95% CI) and p-value for injured and non-injured sides, for the 16 participants with ACL rupture.

SLS and DLS are included in the Test for Substitution Patterns where altered movements (substitution patterns) were scored as observable, predefined deviations of postural orientation; for details see Subjects and Methods section.

### SEMG

**Figure 1:**
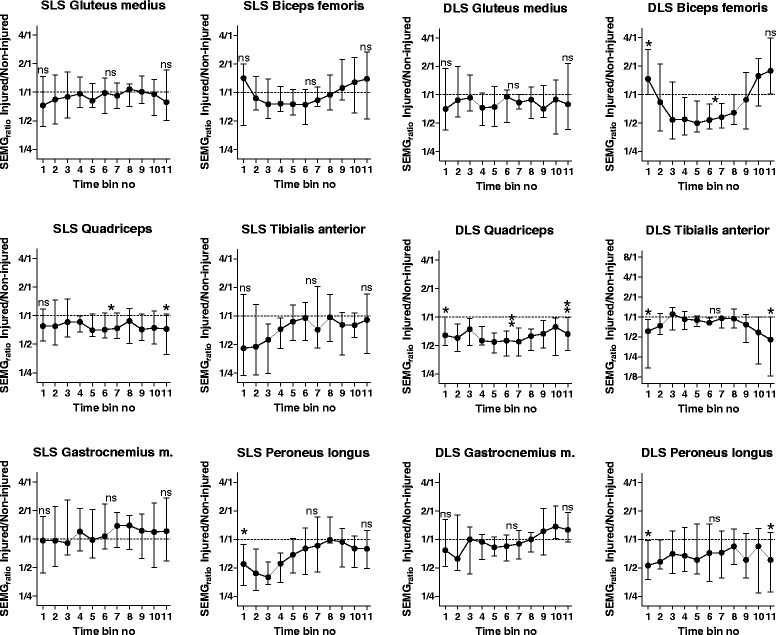
**Median SEMG**_**ratios**_ **for 11 time bins for each muscle for Single- and Double Leg Squat.** Median and quartiles (Q_1_ and Q_3_) of the SEMG_ratios_ for the 16 participants with ACL rupture plotted for the 11 time bins for each muscle for the two test movements Single Leg Squat (SLS) and Double Leg Squat (DLS) with a horizontal line representing equal activity of the injured and non-injured sides. Evaluation of statistical significance were performed for time bins 1, 6/7 and 11, and are indicated in the figure; ns = non-significant, * = p ≤ 0.05. ** = p ≤ 0.01.

In SLS, overall activity in time bins 1, 6/7 and 11 was similar in non-injured and injured sides, with three notable exceptions (Figure 1, Table 3). (For absolute amplitudes of SEMG during entire SLS and DLS performance, see Additional file 1). Prior to the beginning of movement, in bin 1, activity in peroneus longus was lower on the injured side, indicated by a SEMG_ratio_ of 0.55 [Q_1_, Q_3_ 0.33, 0.88] (p = 0.03). At transition from knee flexion to extension, activity in quadriceps vastus lateralis was lower on the injured side; SEMG_ratio_ 0.69 [Q_1_, Q_3_ 0.62, 0.97] (p = 0.05). At end of SLS, there was still a lower activity in quadriceps on the injured side; SEMG_ratio_ 0.73 [Q_1,_ Q_3_ 0.39, 1.05] (p = 0.05).

**Table 3:** **SEMG**_**ratios**_ **for the Single Leg Squat (SLS) and the Double Leg Squat (DLS)**

**SLS**						
Muscle	Time bin 1	p	Time bin 6/7	p	Time bin 11	p
	Median (Q_1_, Q_3_)		Median (Q_1_, Q_3_)		Median (Q_1_, Q_3_)	
Quadriceps vastus lateralis	0.78 (0.54, 1.18)	ns (0.12)	0.69 (0.62, 0.97)	0.05	0.73 (0.39, 1.05)	0.05
Peroneus longus	0.55 (0.33, 0.88)	0.03	0.72 (0.44, 1.36)	ns (0.16)	0.80 (0.49, 1.25)	ns (0.16)
DLS						
Muscle	Time bin 1	p	Time bin 6/7	p	Time bin 11	p
	Median (Q_1_, Q_3_)		Median (Q_1_, Q_3_)		Median (Q_1_, Q_3_)	
Biceps femoris	1.46 (1.00, 3.00)	0.04	0.66 (0.48, 0.80)	0.02	1.78 (1.02, 3.96)	ns (0.11)
Quadriceps vastus lateralis	0.64 (0.50, 1.00)	0.04	0.56 (0.38, 0.71)	0.003	0.66 (0.44, 0.99)	0.01
Tibialis Anterior	0.61 (0.17, 0.92)	0.02	0.82 (0.76, 0.99)	ns (0.15)	0.46 (0.13, 1.00)	0.02
Peroneus longus	0.53 (0.38, 0.98)	0.02	0.70 (0.35, 1.45)	ns (0.13)	0.61 (0.28, 1.19)	0.04

Median SEMG_ratios_, quartiles and p-value for the Single Leg Squat (SLS), and the Double Leg Squat (DLS), for the muscles with statistically significant differences between sides in one or more of the time bins 1, 6/7 and 11, for the 16 participants with ACL rupture.

SEMG_ratio_ was calculated as the ratio of the average amplitude between injured and non-injured sides for each of the time bins 1, 6/7 and 11, so that an equal activation in injured and non-injured sides would correspond to 1.0. The calculations were made for all six muscles recorded.

SLS and DLS are included in the Test for Substitution Patterns where altered movements (substitution patterns) were scored as observable, predefined deviations of postural orientation; for details see Subjects and methods section.

Time bin = a time epoch. Time bin 1 corresponds to the instance prior to the beginning of the movement, time bin 6/7 to the transition from flexion to extension and time bin 11 to the end of the movement.

Q_1_, Q_3_ = quartiles 1 and 3.

p = p-value.

ns = non-significant.

In DLS, activity prior to the beginning of the movement differed between injured and non-injured sides in four of the six muscles recorded (Figure 1, Table 3). The SEMG_ratio_ was 1.46 [Q_1,_ Q_3_ 1.00, 3.00], (p = 0.04) for biceps femoris, 0.64 [Q_1,_ Q_3_ 0.50, 1.00] (p = 0.04) for quadriceps, 0.61 [Q_1,_ Q_3_ 0.17, 0.92] (p = 0.02) for tibialis anterior and 0.53 [Q_1,_ Q_3_ 0.38, 0.98] (p = 0.02) for peroneus longus. At transition from knee flexion to extension, SEMG_ratio_ was 0.66 [Q_1,_ Q_3_ 0.48, 0.80] (p = 0.02) for biceps femoris and 0.56 [Q_1,_ Q_3_ 0.38, 0.71] (p = 0.003) for quadriceps. At the end of DLS there was lower activity on the injured side in three muscles – quadriceps vastus lateralis, SEMG_ratio_ 0.66 [Q_1,_ Q_3_ 0.44, 0.99] (p = 0.01), tibialis anterior, 0.46 [Q_1,_ Q_3_ 0.13, 1.00] (p = 0.02) and peroneus longus, 0.61 [Q_1,_ Q_3_ 0.28, 1.19] (p = 0.04).

The activity in antagonistic muscles, calculated as a ratio of the SEMG activity of quadriceps/hamstrings within the injured side was lower for the SLS on injured side in bin 11, with a median of 1.80 [Q_1,_ Q_3_ 1.07, 2.67], as compared to the non-injured side, with a median ratio of 3.6 [Q_1,_ Q_3_ 2.02, 5.73] and a median difference of 1.73, 95% CI −0.6 to 4.5, p = 0.02. For the DLS there was a lower quadriceps/hamstrings ratio on injured side prior to the beginning of the movement in bin 1, with a median of 1.9 [Q_1,_ Q_3_ 0.73, 3.25], as compared to non-injured side of 4.5 [Q_1,_ Q_3_ 2.92, 6.0] and a median difference of 2.5, 95% CI 0.4 to 4.0, p = 0.02, and at end of DLS in bin 11, with a lower quadriceps/hamstrings ratio on injured side of 1.9 [Q_1,_ Q_3_ 1.02, 3.68], as compared to on the non-injured side, with 4.3 [Q_1,_ Q_3_ 3.52, 7.64] and a median difference of 2.5, 95% CI 0.1 to 5.9, p = 0.03. The only deviation in antagonistic activity on the injured side for the tibialis anterior/gastrocnemius muscles was a lower activity at end of DLS in bin 11, with a median on injured side of 0.3 [Q_1,_ Q_3_ 0.18, 0.73], as compared to the non-injured side, with 1.14 [Q_1,_ Q_3_ 0.54, 5.07] and a median difference of 1.0, 95% CI 0 to 5.3, p = 0.004.

### Correlations between TSP scores and SEMG_ratio_

In SLS, “lateral displacement of the hip-pelvis region on the supporting side” was negatively correlated to SEMG_ratio_ of biceps femoris in time bin 1, r_s_ = −0.71 (p = 0.002, 95% CI −0.89 to −0.33), and to SEMG_ratio_ of quadriceps in time bin 6/7, r_s_ = −0.54 (p = 0.03, 95% CI −0.82 to −0.06). These findings indicate that the lower the muscle activity in biceps femoris on the injured side prior to the beginning of movement and the lower the activity on the injured side in quadriceps at transition from knee flexion to extension, the more pronounced the lateral displacement of the hip-pelvis region (Table 4). “Knee medial to supporting foot” was negatively correlated to SEMG_ratio_ of the gluteus medius in time bin 6/7, r_s_ = −0.73 (p = 0.001, 95% CI −0.90 to −0.36), indicating that the lower the muscle activity in gluteus medius at transition from knee flexion to extension, the more pronounced the displacement of the knee in relation to the supporting foot. “Displacement of trunk” was positively correlated to SEMG_ratio_ for gluteus medius in time bin 6/7, r_s_ = 0.62 (p = 0.01, 95% CI 0.18 to 0.85) and negatively correlated to SEMG_ratio_ for tibialis anterior in time bin 11, r_s_ = −0.50, (p = 0.047, 95% CI −0.80 to −0.01). This indicates that high activity in gluteus medius on the injured side at transition from knee flexion to extension and low activity in tibialis anterior on the injured side at the end of the SLS, were associated with more pronounced displacement of the trunk. Finally, there was a negative correlation between “pronation of foot” and SEMG_ratio_ for tibialis anterior in time bin 11, r_s_ = −0.60, (p = 0.015, 95% CI −0.84 to −0.14), indicating that low activity in tibialis anterior at the end of the movement was associated to a more pronounced pronation of the foot.

**Table 4:** **Correlations between TSP points and SEMG**_**ratios**_ **in Single- and Double Leg Squat (SLS, DLS)**

**SLS**			
Substitution pattern according to TSP	SEMG_ratio_ for muscle	Time bin	r_s_ (95% CI) p-value
“lateral displacement of the hip-pelvis region on the supporting side”	biceps femoris	1	−0.71 (−0.89 to −0.33) 0.002
“lateral displacement of the hip-pelvis region on the supporting side”	quadriceps	6/7	−0.54 (−0.82 to −0.06) 0.03
“knee medial to the supporting foot”	gluteus medius	6/7	−0.73 (−0.90 to −0.36) 0.001
“displacement of the trunk”	gluteus medius	6/7	0.62 (0.18 to 0.85) 0.01
“pronation of the foot”	tibialis anterior	11	−0.60 (−0.84 to −0.14) 0.015
“displacement of the trunk”	tibialis anterior	11	−0.50 (−0.80 to −0.01) 0.047
**DLS**			
“displacement of body weight to either side”	tibialis anterior	11	−0.51 (−0.80 to −0.02) 0.043

Correlations between the points scored for the substitution patterns in injured side and SEMG_ratios_ for the muscles recorded, in the test-movements Single Leg Squat (SLS) and Double Leg Squat (DLS), for the 16 participants with ACL rupture. Spearman’s correlation coefficients, the 95% confidence interval and p-value are presented for time bins 1, 6/7 and 11 with statistically significant correlations.

SEMG_ratio_ was calculated as the ratio of the average amplitude between injured and non-injured sides for each of the time bins 1, 6/7 and 11, so that an equal activation in injured and non-injured sides would correspond to 1.0. The calculations were made for all six muscles recorded.

SLS and DLS are included in the Test for Substitution Patterns where altered movements (substitution patterns) were scored as observable, predefined deviations of postural orientation; for details see Subjects and methods section.

Time bin = a time epoch. Time bin 1 corresponds to the instance prior to the beginning of the movement, time bin 6/7 to the transition from flexion to extension and time bin 11 to the end of the movement.

r_s_ = Spearman’s correlation coefficient.

95% CI = 95% confidence interval.

For DLS, “displacement of body weight to either side” was negatively correlated to SEMG_ratio_ for tibialis anterior in time bin 11, r_s_ = −0.51 (p = 0.043, 95% CI −0.80 to −0.02), indicating that low activity in tibialis anterior at the end of the movement, was associated to a more pronounced displacement of body weight (Table 4).

## Discussion

More pronounced substitution patterns were observed on participants’ injured side, in agreement with previous studies of the TSP [3,9]. Deviating muscular activity, in general lower activity on the injured as compared to the non-injured side, and deviations between injured and non-injured side in antagonistic activity within the injured side were found during both SLS and DLS. Correlations between individual substitution patterns and deviations in the activity of specific muscles at specific times were seen, not only in muscles acting directly on the knee joint, but also on adjacent joints. These results support the notion that specific altered movement patterns may be associated with deviations in muscular activity.

The SEMG_ratio_ was calculated for six muscles at three time bins (time bin 1, 6/7 and 11) during SLS and DLS, amounting to 18 comparisons between injured and non-injured sides for each movement. Out of these 18 comparisons for SLS and DLS respectively, 3 for SLS and 9 for DLS showed statistically significantly differences (Table 3). With only one exception, all these differences constituted a lower activity on the injured as compared to the non-injured side prior to beginning of movement, at transition from flexion to extension as well as at the end of movement. In DLS, a significantly lower activity was seen in four of six muscles prior to the beginning of movement, two of six muscles in transition from flexion to extension and in three of six muscles at end of movement. Notably, quadriceps had a significantly lower activity on the injured side in all three phases of the movement. A lower activity in quadriceps muscle at transition from flexion to extension and at end also was found during SLS. A key to understanding mechanisms underlying altered movement patterns will be to determine if changes in muscular activation such as those described here occur as a direct consequence of ACL injury, or whether they are adaptive and act to compensate for loss of joint stability and/or sensory afferent input. While such analysis of causality was beyond the scope of the present study, a first step may be to put our results in perspective of previous findings.

One reason for the low injured/non-injured ratios at transition from knee flexion to extension in quadriceps in SLS and DLS can be the proposed arthrogenic muscle inhibition in quadriceps, describing the inability to contract the muscle after joint injury beyond conscious, voluntary control [36]. This is found to be common both in individuals with ACL-injury and ACL-reconstruction, often observed bilaterally [37]. Although muscle inhibition was not assessed in the individuals in the present study, it is likely that the occurrence of quadriceps inhibition could explain part of the low injured/non-injured ratios in quadriceps found in the individuals studied in this study.

Another reason for a decrease in muscle activity on the injured side could be muscle weakness, often reported after ACL injury, especially in quadriceps and hamstrings [38] in which a quadriceps strength deficit of 20% preoperatively and also 1 year after ACL reconstruction is reported [39]. EMG amplitude reflects to some degree muscle force development in the non-fatigued state [40]. When comparing EMG amplitudes between muscles or individuals, it is common to normalize the values against the EMG amplitude recorded at maximal voluntary contraction (MVC). We chose not to perform MVCs due to the relatively short time that had passed after the ACL injury, since MVCs could endanger instability in the injured knee joint, with the associated risk of subsequent pain or re-injury to the affected knee but also a risk of pain during the performance of MVCs that could affect the assessment itself. Therefore, no information on muscle force development in our participants was available. Thus, it cannot be excluded that muscle weakness can partly reflect the lower EMG activity on the injured side, especially when all loading is concentrated on one leg, as in the SLS. Whether such assumed muscle weakness was present before injury or occurred as a (direct or adaptive) consequence of injury is not known. However, since scores on the KOOS for this group of participants were similar to individuals with ACL-injury assigned for ligament reconstruction the tasks performed in the present study may be argued to be submaximal and well within participants’ capacity. More importantly, the results of the deviating antagonistic activity patterns with a lower quadriceps/hamstrings ratio within injured as compared to within non-injured sides prior to the beginning of DLS movement, at end of both SLS and DLS, and for the tibialis/gastrocnemius ratio at end of DLS, assessed in phases of the movement in which muscle strength is not challenged, cannot be explained only in terms of muscle weakness, but further point towards an altered muscular activation pattern affecting sensorimotor control and therefore most likely contributing to the altered movement patterns. These discrepancies may be an important factor underlying the development of substitution patterns, and should be further investigated in future studies.

The only muscle with higher activity on the injured as compared to non-injured side was biceps femoris just prior to the beginning of DLS movement. The hamstrings and the ACL are postulated to act in synergy to prevent anterior tibial displacement during quadriceps contraction and it also has been suggested that increased hamstring activity and quadriceps inhibition may occur as a reactive muscle strategy to regain functional stability [41]. However, investigations of hamstrings muscle activity show varying results. During gait, Boerboom et al. reported high hamstrings activity in the beginning and at the end of a stride in both individuals with ACL injury and in controls [42], while Alkjaer et al. [43], found no differences in EMG patterns of hamstrings muscles when comparing individuals with ACL injury to controls. Swanik et al. found increased hamstrings activity during landing from a jump in individuals with ACL injury and concluded that this reactive muscle activity was presumably an attempt to regain functional stability [41]. The reported changes in biceps femoris muscle activity, as found prior to the beginning of DLS movement in the present study and in accordance with Swanik et al. [41], might indicate adaptive changes in motor control as a strategy to regain functional stability. If so, this could be speculated to be a result of changes in sensory feedback due to the injured ligament, and a modification in existing motor programs by regaining or relearning new movement patterns. Together with the results of the deviating antagonistic activity patterns of quadriceps/hamstrings- and tibialis/gastrocnemius ratio within the injured as compared to the non-injured sides in the present study, adaptive changes in sensorimotor control certainly cannot be excluded.

When performing DLS, it is possible to shift more loading to the non-injured side, which could explain the lower SEMG_ratio_ on the injured side. This also was indicated by the scoring of the TSP, since “displacement of body weight to either side” was only observed on the injured side. The mechanism underlying the displacement is unclear, but one plausible explanation may be the experience of pain at time of injury resulting in a subsequent fear of movement. Individuals with ACL injury may have lower muscle strength on the injured side even after one year of rehabilitation [38]. This decreased strength might be affected by habitual displacement of bodyweight or limited attention in rehabilitation programs to weight bearing. Therefore, it would be important to make the individual aware of habitual displacement of body weight and to address this in training of sensorimotor control, strength and endurance through augmented visual feedback using mirrors, or biofeedback.

We found correlations between low SEMG_ratio_ for gluteus medius and more pronounced substitution patterns of “knee medial to supporting foot” during SLS. This is in accordance with Mauntel et al., who found associations between medial knee displacement and decreased co-activation of gluteal to hip adductor muscles in non-injured individuals performing SLS [28]. In addition, we found correlations between low SEMG_ratio_ for quadriceps muscle and more pronounced “lateral displacement of hip-pelvis region”. These findings, together with findings of substitution patterns in the whole lower extremity and trunk as measured with the TSP in previous studies [3,9], and by analogy with the functional tests used by Whatman et al. rating trunk, pelvis, knee and foot [18], indicate that the whole kinetic chain should be observed in assessment of altered movement patterns.

Some limitations should be considered when the findings of this study are interpreted. The amplitude and frequency content of the SEMG signal, as well as the susceptibility to crosstalk from adjacent muscles, is primarily dependent on the distance between the electrodes and the active muscle fibers. Electromagnetic interference and movement artefacts may disturb the signal, and contraction velocity, muscle length, tissue thickness, temperature etc. also can influence the signal, why these factors have to be controlled and minimized when comparisons between individuals, muscles or sessions are performed [44]. Since all comparisons were conducted between injured and non-injured sides in the same individual on one occasion, the above mentioned variations were kept to a minimum. Since the movement speed can influence SEMG amplitude [45], a metronome was used to diminish differences between sides and since the same examiner mounted all electrodes, the variation in electrode application and placement was kept to a minimum.

In accordance with previous work we found noticeable variations in absolute amplitudes between individuals, Additional file 1 [46]. By calculating SEMG_ratio_ these differences were diminished. Yet, the low amplitudes, especially in time bin 1 and 11, in which a small change in amplitude can induce considerable change in ratio, might imply some uncertainty in the results. Therefore, SEMG_ratios_ calculated for higher amplitudes, as in bin 6/7 with transition from flexion to extension, had a stronger influence on our conclusions.

Another aspect not yet investigated is if altered movement patterns and muscular activity are dissimilar in different subgroups, for example if divided into subgroups of men and women. Diminished muscular protection of passive structures of the knee have been described in women as compared to men [47,48], and Myer et al. have suggested that females utilize neuromuscular activation strategies which may contribute to “dynamic valgus” when performing high-risk manoeuvres [49]. However, since the present study was a first evaluation of muscle activity during altered movement patterns scored with TSP, a distribution into subgroups of men and women was neither achievable, nor within the scope of the study, but ought to be investigated in future studies.

## Conclusions

More pronounced altered movement patterns were observed on participants’ injured than on non-injured side. Deviations in SEMG activity of specific muscles between injured and non-injured sides were seen during both SLS and DLS – with generally lower activity on the injured side. Associations between these deviations and specific altered movement patterns were found, particularly during SLS. This was found not only for muscles acting directly across the knee joint, but also for the gluteus medius muscle. Also, deviating antagonistic muscular activity patterns in quadriceps/hamstrings and in tibialis/gastrocnemius within injured as compared to non-injured sides were found. These findings suggest that altered movement patterns during functional assessments and rehabilitative exercise such as the SLS and DLS – more strenuous than gait, but not as demanding as jumping – are not only caused by altered biomechanics and/or muscle weakness but probably also by altered sensorimotor control. We suggest that in the evaluation and rehabilitation of ACL injury, assessments of specific altered movement patterns, using for instance the TSP, might provide indications of specific alterations in sensorimotor control and therefore should be considered as a complement to other assessments.

## Additional file

Additional file 1:
**Absolute amplitudes Trulsson et al. 2014.** Absolute amplitudes. Median and quartiles (Q_1_ and Q_3_) for absolute amplitudes for each time bin, each muscle, for non-injured and injured sides for the test-movements Single Leg Squat (SLS) and the Double Leg Squat (DLS) for 16 participants with ACL rupture.
