# Supplementary material for: Altered movement patterns and muscular activity during single and double leg squats in individuals with anterior cruciate ligament injury
Source: BMC Musculoskelet Disord. 2015 Feb 13;16:28. doi: 10.1186/s12891-015-0472-y (PMC4333170; doi:10.1186/s12891-015-0472-y)

Additional file 1

Title: Absolute amplitudes

Median and quartiles (Q_1 and Q_3) for absolute amplitudes for each time bin, each muscle, for non-injured and injured sides for the test-movements Single Leg Squat (SLS) and the Double Leg Squat (DLS) for 16 participants with ACL rupture.

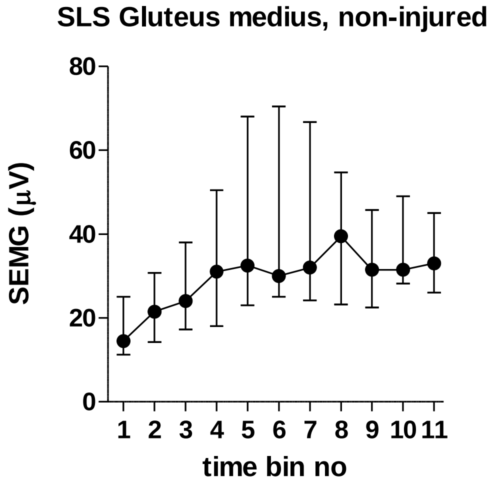
SLS Gluteus medius, non-injured
SEMG (µV)
0
20
40
60
80
1 2 3 4 5 6 7 8 9 10 11
time bin no

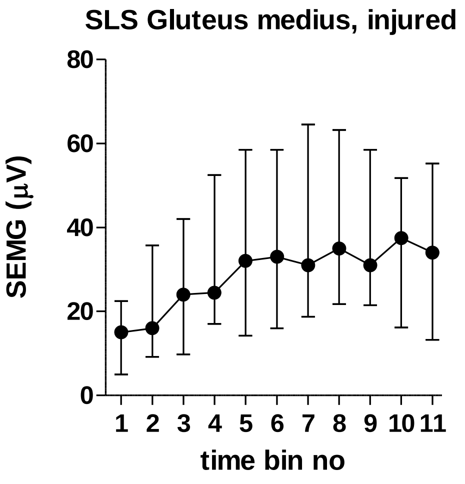
SLS Gluteus medius, injured
SEMG (µV)
0
20
40
60
80
1 2 3 4 5 6 7 8 9 10 11
time bin no

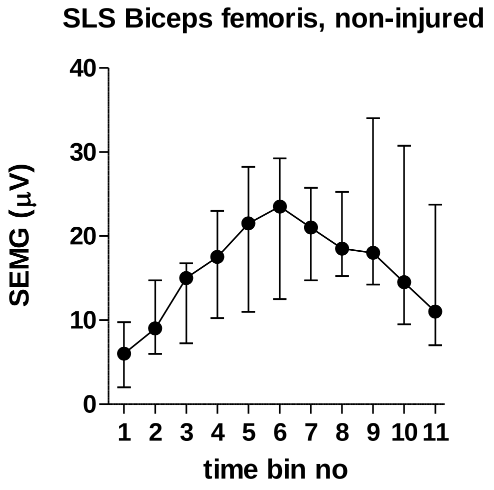
SLS Biceps femoris, non-injured
SEMG (µV)
0
10
20
30
40
1 2 3 4 5 6 7 8 9 10 11
time bin no

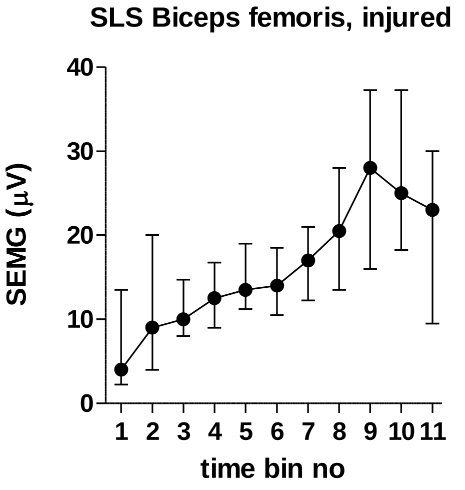
SLS Biceps femoris, injured
SEMG (µV)
0
10
20
30
40
1 2 3 4 5 6 7 8 9 10 11
time bin no

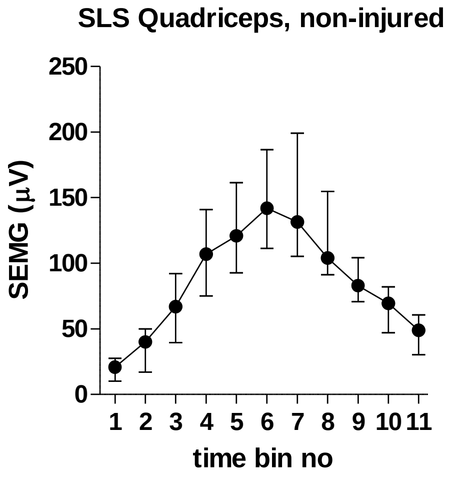
SLS Quadriceps, non-injured
SEMG (µV)
0
50
100
150
200
250
1 2 3 4 5 6 7 8 9 10 11
time bin no

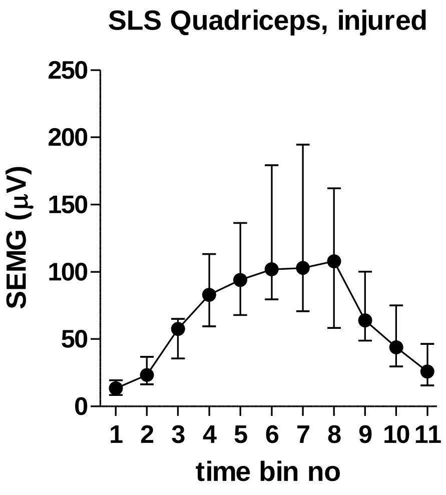
SLS Quadriceps, injured
SEMG (µV)
0
50
100
150
200
250
1 2 3 4 5 6 7 8 9 10 11
time bin no

SLS Tibialis anterior, non-injured

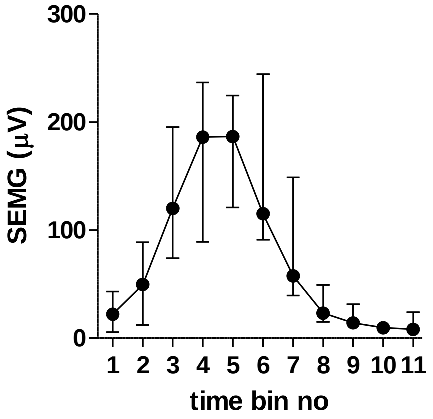

SLS Tibialis anterior, injured

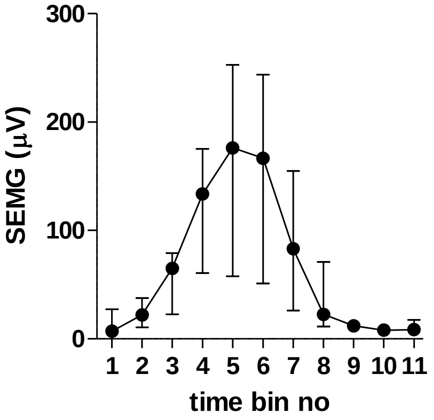

SLS Gastrocnemius m., non-injured

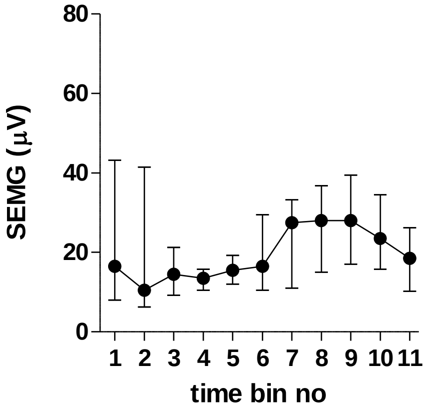

SLS Gastrocnemius m., injured

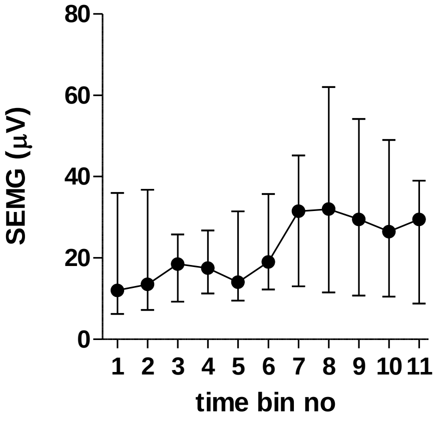

SLS Peroneus longus, non-injured

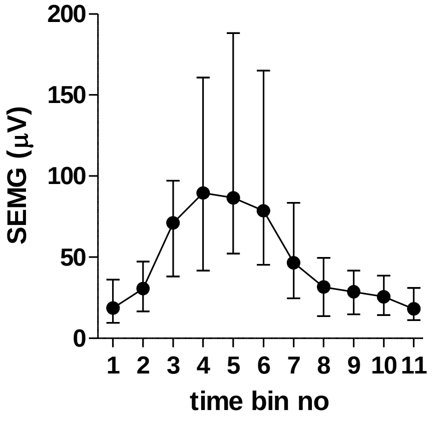

SLS Peroneus longus, injured

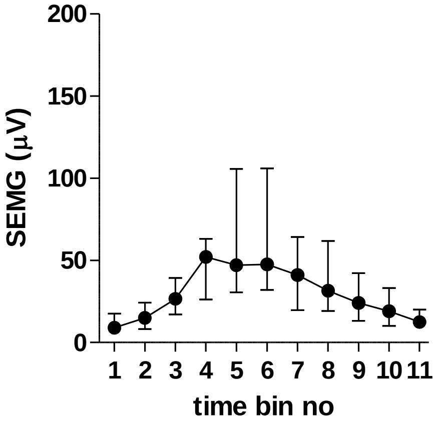

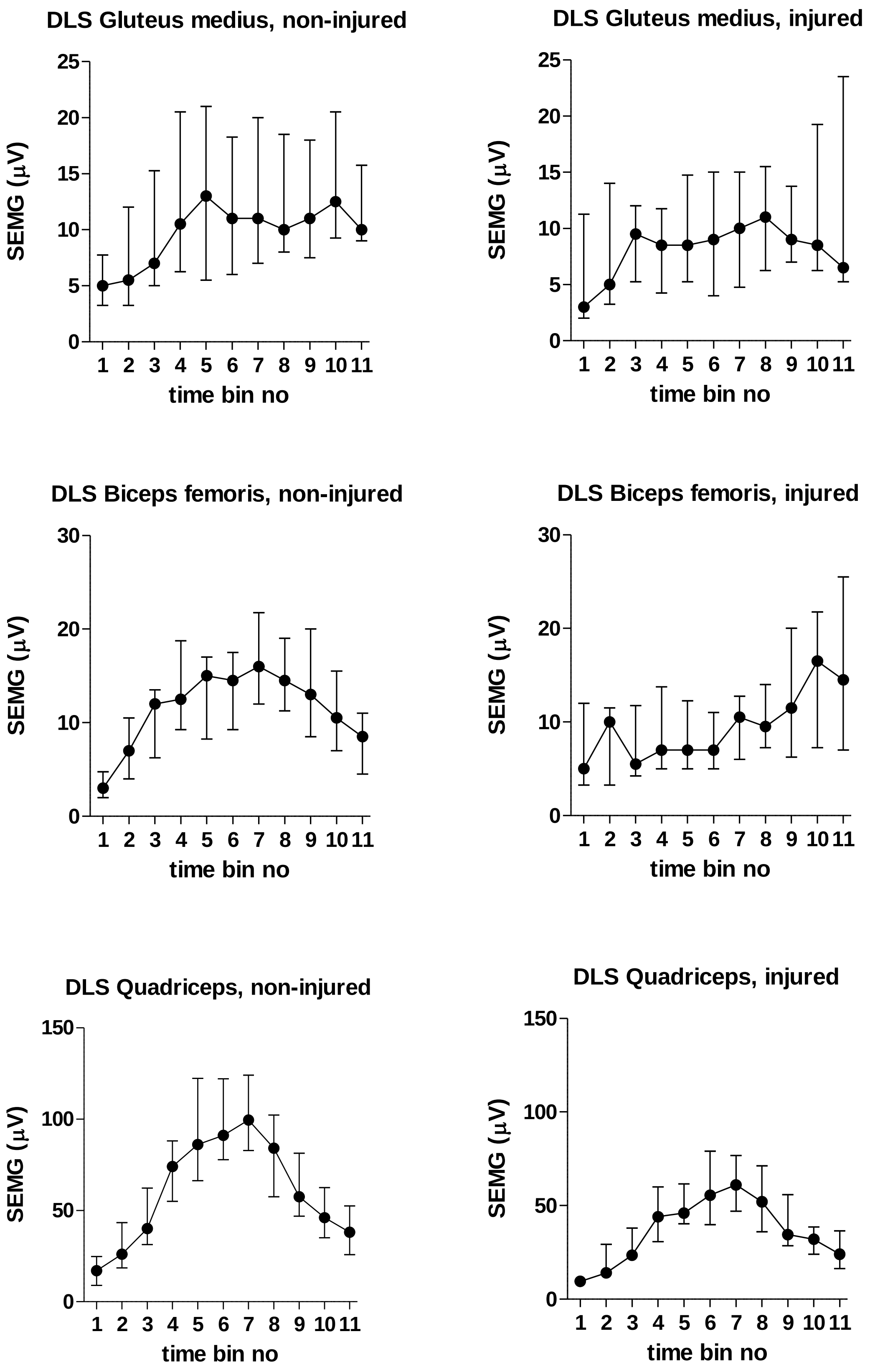
DLS Gluteus medius, non-injured
DLS Gluteus medius, injured
DLS Biceps femoris, non-injured
DLS Biceps femoris, injured
DLS Quadriceps, non-injured
DLS Quadriceps, injured
SEMG (μV)
time bin no
0
5
10
15
20
25
30
50
100
150
1 2 3 4 5 6 7 8 9 10 11

DLS Tibialis anterior, non-injured

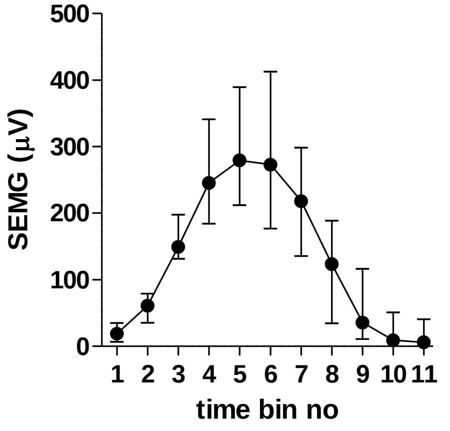

DLS Tibialis anterior, injured

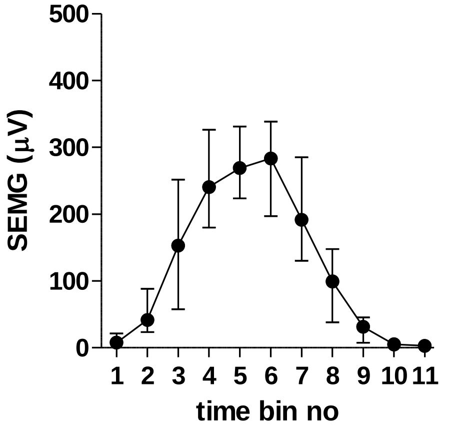

DLS Gastrocnemius m., non-injured

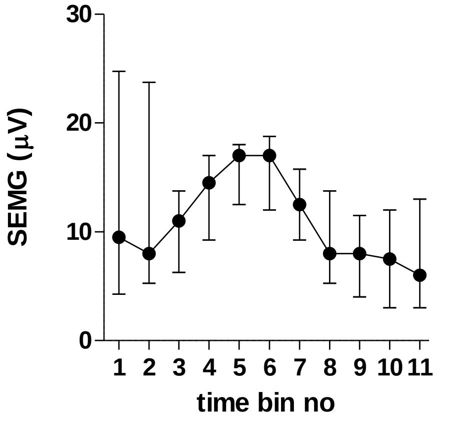

DLS Gastrocnemius m., injured

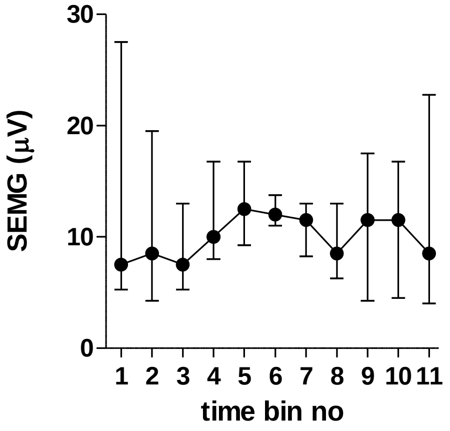

DLS Peroneus longus, non-injured

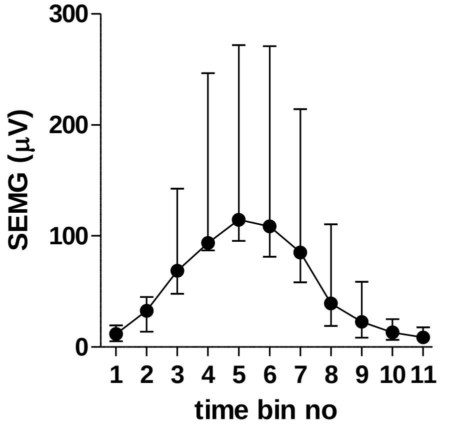

DLS Peroneus longus, injured

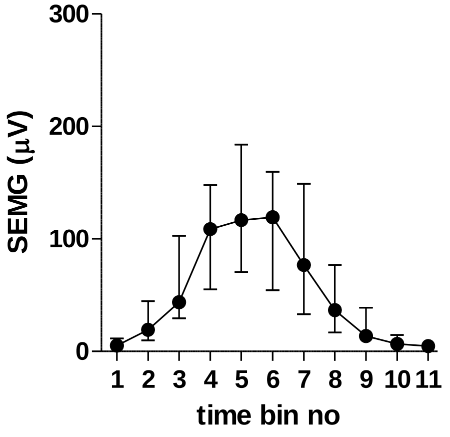

Supplement: Additional file 1: — Absolute amplitudes Trulsson et al. 2014. Absolute amplitudes. Median and quartiles (Q1 and Q3) for absolute amplitudes for each time bin, each muscle, for non-injured and injured sides for the test-movements Single Leg Squat (SLS) and the Double Leg Squat (DLS) for 16 participants with ACL rupture. [file 12891_2015_472_MOESM1_ESM.pdf]
